# Advance in Detection Technique of Lean Meat Powder Residues in Meat Using SERS: A Review

**DOI:** 10.3390/molecules28227504

**Published:** 2023-11-09

**Authors:** Qinghui Guo, Yankun Peng, Jianwei Qin, Kuanglin Chao, Xinlong Zhao, Tianzhen Yin

**Affiliations:** 1 College of Engineering, China Agricultural University, Beijing 100083, China; 2 College of Biosystems Engineering and Food Science, Zhejiang University, Hangzhou 310058, China; 3 USDA/ARS Environmental Microbial and Food Safety Laboratory, Beltsville Agricultural Research Center, 10300 Baltimore Ave., Beltsville, MD 20705, USA

**Keywords:** surface–enhanced Raman spectroscopy, lean meat powder, livestock and poultry meat, residues, nanomaterials

## Abstract

Food that contains lean meat powder (LMP) can cause human health issues, such as nausea, headaches, and even death for consumers. Traditional methods for detecting LMP residues in meat are often time-consuming and complex and lack sensitivity. This article provides a review of the research progress on the use of surface–enhanced Raman spectroscopy (SERS) technology for detecting residues of LMP in meat. The review also discusses several applications of SERS technology for detecting residues of LMP in meat, including the enhanced detection of LMP residues in meat based on single metal nanoparticles, combining metal nanoparticles with adsorbent materials, combining metal nanoparticles with immunizing and other chemicals, and combining the SERS technology with related techniques. As SERS technology continues to develop and improve, it is expected to become an even more widely used and effective tool for detecting residues of LMP in meat.

## 1. Introduction

Lean meat powder (LMP) is a synthetic *β*-receptor agonist whose full name is beta-adrenoceptor agonist (*β*-adrenoceptor agonist), also known as a beta stimulant [1,2]. Due to its broncho–dilatory activity, it is widely used in medicine to treat human respiratory diseases such as obstructive pneumonia, smooth muscle spasms, and bronchial asthma [3,4]. LMP is not only effective in increasing feed conversion but also promotes animal growth and fat burning and significantly improves carcass quality [5,6]. Research has shown that feeding livestock with LMP can increase the lean meat rate by 9% to 16% and reduce skeletal muscle fat by 8% to 15% [7,8]. As a result, LMP has been used as a feed additive by some farmers and farming companies for raising livestock, and pigs fed on this feed are also known as “lean-meat pigs”. When illegal merchants use LMP to feed livestock, the amounts of the additive are usually larger [9]. Since there is no stopping period for drugs before slaughter, there will be a high concentration of LMP residue in the muscle and internal organs, and it is not easy to decompose [10,11,12]. Food contaminated with LMP can cause human health issues such as dizziness, kidney damage, vomiting, muscle pain, and even death [13,14,15].

Therefore, for the sake of food safety, many countries, including the European Union and China, have established rigorous regulations banning the addition of LMP to animal feed [16,17]. Although ractopamine is allowed in the United States and Canada, a maximum residue limit of 10 µg/kg of ractopamine in meat has been established [18,19]. Despite the issuance of several regulations, some unscrupulous merchants continue to use LMP to feed livestock for profiteering. In 2017, Russia decided to ban the import of beef from New Zealand because ractopamine residues were detected in the beef. In 2021, the “tainted lamb” incident in Cangzhou, Hebei, China was reported. Therefore, the regulation and control of LMP residues in meat and animal feed should be strengthened to ensure food safety.

Currently, the detection techniques for LMP residues in animal feed and food include high-performance liquid chromatography (HPLC) [20,21], liquid chromatography-mass spectrometry (LC–MS) [22,23], gas chromatography-mass spectrometry (GC–MS) [24], ultra-high-performance liquid chromatography (UHPLC) [25], and capillary electrophoresis (CE) [26], etc. Although the above methods have lower limits of detection and better stability, they have the disadvantages of long detection time, expensive instruments, and complicated operation. Therefore, there is an urgent need for a rapid, accurate, and efficient detection method to realize the rapid detection of LMP in livestock and poultry meat.

Surface-enhanced Raman scattering (SERS) is a technique that enhances the Raman scattering strength of a target molecule by adsorbing it on the surface of a metal nanoparticle [27,28,29]. SERS technology can increase the Raman signal intensity of target molecules by 10^6^–10^14^ times, which is characterized by high sensitivity, fast detection speed, low detection cost, easy operation, and portability [30,31]. Currently, SERS technology has been successfully applied in the fields of environment, public safety, bioanalysis, pollutant monitoring, and food safety [32,33,34,35]. In recent years, SERS technology has made great advances in the trace analysis and detection of banned or limited-use chemicals such as pesticides, food additives, antibiotics, etc. [36,37].

There have been more reports on the application of SERS technology in agriculture and food testing. The advances in SERS sensor research on contaminants in water treatment were discussed by Barbillon et al. [38]. An overview of the characteristics of different SERS sensors and their applications in agriculture and related fields was reported by Liu et al. [39]. The application of nano-sensors for the detection of clenbuterol in food samples, focusing on a wide range of techniques for the detection of clenbuterol, was reviewed by Jigyasa et al. [40]. The above reviews have a different focus from this paper. In this review, the types, mechanism of action for LMP, and the principles of SERS technology are described. A comprehensive discussion is then presented on the progress of research based on single metal nanoparticles, metal nanoparticles in combination with adsorbent substances and chemicals as enhancement substrates, and SERS technology in combination with other techniques for detecting the residues of LMP in meat. Finally, the current research status and future perspectives of SERS technology for the detection of LMP in meat are summarized.

## 2. Physical and Chemical Properties of LMP

### 2.1. Types of LMP

LMP mainly includes drugs such as clenbuterol, ractopamine, salbutamol, cimaterol, clorprenaline, terbutaline, tulobuterol, penbutolol, brombuterol, and their salts and lipids [41]. The most used feed additives are clenbuterol, ractopamine, and salbutamol and their salts and lipids.

### 2.2. Molecular Structure of LMP

**Figure 1:**
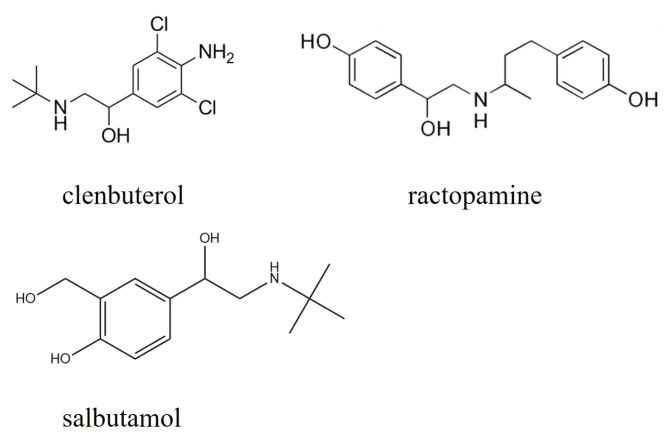
Molecular structure of selected LMP drugs.

Among the LMP drugs, clenbuterol, ractopamine, and salbutamol are widely used, and their molecular structures are shown in Figure 1. As shown in Figure 1, all three drug molecules have a benzene ring structure and numerous nonpolar chemical bonds, which provide the basis for Raman detection.

### 2.3. Mechanisms of Action

Some studies suggest that *β*-agonists may decrease lipid synthesis and increase lipolysis through insulin-related mechanisms [42]. Specifically, the LMP drug can act on the *β*-adrenergic receptors in the bodies of live animals and activate adenylate cyclase in the body. Adenylate cyclase can produce cyclic adenosine monophosphate (cAMP), which in turn regulates the activity of intracellular enzymes, inhibits glycogen synthesis, promotes fat catabolism, and ultimately achieves the goal of reducing body fat [43].

## 3. Raman Spectroscopy and Surface-Enhanced Raman Scattering Techniques

### 3.1. Raman Spectroscopy

**Figure 2:**
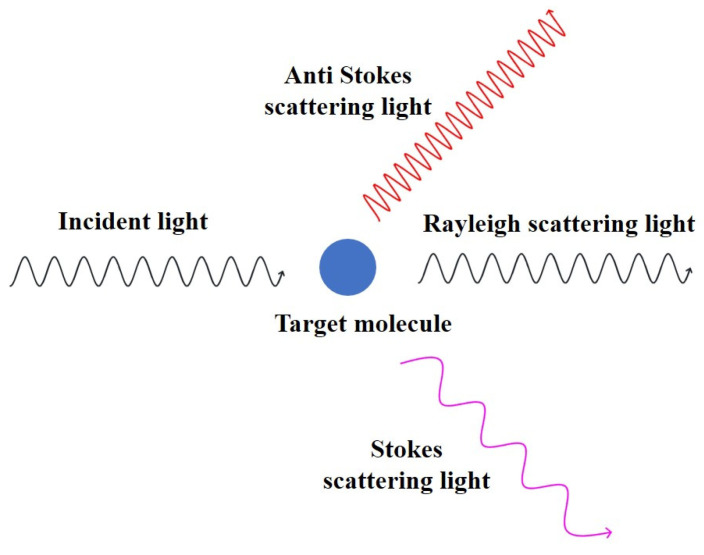
The action of light on molecules.

As shown in Figure 2, when monochromatic light is irradiated onto a molecule to be measured, a part of the incident light will be absorbed, a part of it will be reflected, and a part of it will be scattered by the action of the molecule [44,45]. Scattered light is further divided into elastic and inelastic scattering [46]. Most of the scattered light coincides with the frequency of the incident light, and this scattered light is known as elastic or Rayleigh scattering [47,48]. A very small fraction (about 1/10^7^ of the scattered light) of the excitation light changes in energy and frequency upon collision with a molecule, which is called inelastic scattering. In inelastic scattering, the vast majority of the energy is diminished, i.e., the frequency of the incident light decreases, and this portion of the light is known as Stokes Raman scattering [49]. A very small portion of the incident light increases in energy, i.e., the frequency of the incident light increases, and this portion of the light is known as anti-Stokes Raman scattering. Stokes Raman scattering is usually measured because it is much more numerous and easier to measure than anti-Stokes Raman scattering. The absolute value of the decrease in the frequency of the incident light is expressed as a Raman shift. The magnitude of the Raman shift is only related to the structure of the molecule itself and is not affected by the frequency of the incident light [50].

### 3.2. Surface-Enhanced Raman Scattering Spectroscopy

In 1974, Fleischmann et al. first found that the Raman signal of pyridine on the surface of a rough silver electrode was enhanced by an order of magnitude of 10^6^, which laid the foundation for the proposal of surface-enhanced Raman scattering (SERS) [51]. Subsequently, Jeanmaire et al. carried out a systematic experimental study and theoretical calculations of the enhancement effect associated with rough surfaces of precious metals, such as gold, silver, and copper, and termed it the SERS effect [52]. SERS is a novel analytical technology for the ultrasensitive detection of nanostructured precious metals based on enhanced local electromagnetic fields near their surfaces [53,54]. It can be combined with detailed qualitative information for trace-level detection, giving a characteristic response for each molecule, which is a true fingerprint of a compound [55,56].

The mechanism of the SERS effect has been controversial, and there are two main enhancement mechanisms recognized by researchers: electromagnetic enhancement (EM) and chemical or charge-transfer enhancement (CM) [57,58].

#### 3.2.1. Enhancement Mechanism of EM

SERS is widely recognized as an enhancement effect caused by EM. In the EM mechanism, when the incident light irradiates on the surface of the noble metal nanoparticles, the surface electrons of the noble metal nanoparticles experience collective oscillation, i.e., surface plasmon resonance (SPR) [59]. This process leads to a redistribution of the local field, where a substantial enhancement of the electromagnetic field (“hot spot”) occurs at a specific location around the nanoparticles, and molecules adsorbed or close to the “hot spot” will have a huge enhancement compared to molecules at other locations. This enhancement is also characterized by a clear distance dependence: only molecules on or extremely close to the metal surface can obtain a great enhancement [60]. The signal of SERS can be maximized when both the incident light as well as the scattered signal of the molecule resonate with the plasma exciton frequency. In addition, EM is non-chemically selective, so it can provide enhancement for any type of molecule.

#### 3.2.2. Enhancement Mechanism of CM

CM refers to the interaction between the molecule to be measured and the metal surface [61,62]. There are currently two main interpretations: the first is that molecule–surface interactions can induce new charge-transfer intermediates with a larger Raman scattering cross-section compared to analytes not adsorbed on the surface. In the second case, when the lowest unoccupied molecular orbital and the highest occupied molecular orbital of a chemisorbed molecule fall symmetrically in the vicinity of the nanoscale energy level on a metal surface, only half the energy of the excitation is required for the jump to occur. Thus, charge transfer between the adsorbent and the metal surface can generate Raman excitation photons [63,64].

In summary, we can conclude that EM enhancement emphasizes the effect of the Raman substrate material on the remote electromagnetic field, which depends on the intrinsic properties of the material such as its size, shape, and composition of the material elements. CE, on the other hand, is achieved by changing the scattering cross-section of the analyte adsorbed on the metal surface, and thus, CE enhancement depends mainly on the chemical properties of the analyte itself. The combination of these two mechanisms creates the SERS mechanism.

### 3.3. Procedure for the Detection of LMP Residues in Meats by SERS Technology

The detection of LMP residues in meat using the SERS technique mainly includes the following steps: preparation of metal nanoparticles, extraction of LMP from pork, acquisition of SERS spectra, and quantitative analysis of the mass fraction of LMP residues in pork using the SERS spectra.

**Figure 3:**
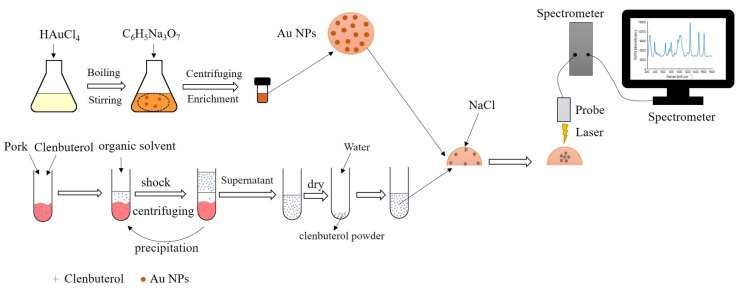
Schematic diagram of the process of detecting clenbuterol in pork by SERS technology.

The synthesis of metal nanoparticles includes ordinary single metal nanoparticles of various shapes and core–shell nanoparticles of multi-metal combinations. The preparation of metal nanoparticles is the key to enhancing the detection of target molecules. A schematic of the process of clenbuterol detection in pork using gold nanoparticles as an enhancement substrate is shown in Figure 3. Gold nanoparticles are first prepared by reducing chloroauric acid using trisodium citrate and then concentrated by centrifugation to obtain a high concentration of gold nanoparticles. Afterward, the clenbuterol in the spiked pork is extracted using an organic solvent and redissolved into ultrapure water. For the detection, a certain volume of gold nanoparticles, the solution to be tested, and the aggregating agent (NaCl) are added. Finally, a Raman spectrometer is used to obtain SERS spectra.

Due to the interference of impurities in the liquid, fluorescence background, baseline drift, droplet morphology changes, etc., the acquired Raman spectra need to be preprocessed using various chemometric methods. Models are then built to quantitatively predict the concentration of the target molecule. Common preprocessing methods include Savitzky–Golay (SG), standardization of variables (SNV), multivariate scattering correction (MSC), adaptive iteratively reweighted penalized least squares, (airPLS), eighth-order polynomial fitting (8th PF), and automatic Whittaker filter (AWF), etc. [65]. Common modeling methods include unary linear regression analysis (ULR), multiple linear regression analysis (MLR), partial least squares regression (PLSR), principal component regression (PCR) and partial least squares–discriminant analysis (PLS–DA), support vector machine (SVM), convolutional neural networks (CNNs), etc. [66].

## 4. Applications for LMP Detection

In this review, research papers on the detection of LMP using SERS technology published on ScienceDirect and Web of Science over the past 15 years are summarized. The details of research papers focused on detecting residues of LMP in meat by SERS, including the limit of detection (LOD) and the enhanced substrates used, are shown in Table 1.

### 4.1. Raman Spectroscopy without Enhancement

Raman spectroscopy without enhancement only allows for the detection of LMP in solid pure form. Raman signals could not be detected in aqueous solutions of LMP at low concentrations. Therefore, only a few research papers have analyzed the Raman characteristic bands of LMP and their attribution.

Ali et al. reported for the first time the Raman spectra of terbutaline, and the corresponding chemical bonds were assigned to the characteristic bands, which provided a reference for the identification and classification of terbutaline [102]. Subsequently, the team investigated the detailed vibrational spectra of salbutamol using mid-infrared and near-infrared Fourier transform (NIR-FT) Raman spectroscopy, and the chemical bonds attributed to the characteristic bands of the Raman spectra were assigned computationally [103].

### 4.2. Application of SERS Spectroscopy for Detecting LMP Residues in Meat

#### 4.2.1. Common Metal Nanoparticle Substrates

Common metal nanoparticles are mainly gold, silver, copper, and aluminum nanoparticles. The type, shape, size, and pH of metal nanoparticles; target molecule extraction method; enrichment method, and type of aggregating agent have a large impact on the enhancement effectiveness of SERS.

In 2011, the detection of clenbuterol in mouse meat using mid-infrared spectroscopy and Raman spectroscopy at wavelengths of 400–3500 cm^−1^ was reported by Meza-Marquez et al., and the PLSR model was established between the mass fraction of clenbuterol in mouse meat and the intensity of characteristic bands. The results showed that the coefficients of determination for modeling using the mid-infrared and SERS methods were 0.996 and 0.914 with standard errors of 0.27 and 1.167, respectively [67]. The effect of particle shape on the enhancement effectiveness of salbutamol was explored by Pham et al. Various shapes of silver nanoparticles were prepared by etching spherical silver nanoparticles. Different shapes of silver nanoparticles were used as the substrate for the detection of salbutamol standard solution with an LOD of 1.25 ng/µL, which were better than the spherical silver nanoparticles [68]. The enhancement effectiveness of gold nanoparticles on clenbuterol, salbutamol, and terbutaline at different pH values was investigated by Lorenzo et al. Clenbuterol, salbutamol, and terbutaline aqueous solutions were detected at optimal pH with LODs of 0.035 mg/L, 0.765 mg/L, and 0.055 mg/L, respectively [69]. Since the characteristic fingerprint of a target molecule was represented by Raman spectra, the technique can be used to classify many different target molecules. Gold nanoparticles were used as an enhancement substrate for the detection and identification of clenbuterol and ractopamine in pork by Zhao et al. After obtaining the Raman spectra of clenbuterol and ractopamine, air-PLS was chosen to remove the fluorescence background information of the spectra. Then, wavelet transform and LSSVM were used to establish a prediction model for the presence or absence of clenbuterol and ractopamine in pork with a better prediction result [70]. The above studies have shown that the detection of LMP in aqueous solutions can be performed using a single metal nanoparticle with an LOD up to the level of mg/L.

Detecting the residues of LMP in meat is generally carried out by extraction, where LMP is extracted and redissolved into an aqueous solution or directly in an organic solvent. Gold nanoparticles were used as an enhanced sinker to detect *β*-agonists such as clenbuterol, salbutamol, and ractopamine in standard solutions. After obtaining the SERS spectra of the three solutions separately, a PLS model was established between their intensity values at the characteristic bands and the corresponding concentrations of the drugs. The model had an LOD of 2 mg/L for clenbuterol and salbutamol standard solutions and 0.1 mg/L for ractopamine. Subsequently, liquid–liquid extraction was used for the rapid extraction and detection of spiked ractopamine in swine urine with an LOD of up to 1 mg/L [71]. In the following year, the group compared three different extraction methods for extracting ractopamine from porcine urine, simple separation, liquid–liquid extraction, and a more complex liquid–liquid extraction combined with solid-phase extraction, and gold-plated Klarite (Renishaw Diagnostics Ltd., Glasgow, UK) was chosen as the substrate for collecting the SERS spectra of ractopamine. The results showed that the Raman characteristic band signal of ractopamine could not be detected by a simple centrifugation method. Instead, the LODs of ractopamine in porcine urine were 0.8 mg/L and 0.4 mg/L by adopting liquid–liquid extraction and liquid–liquid extraction combined with solid-phase extraction, respectively [72]. The LOD was further reduced. Feng et al. compared different extractants for the extraction of spiked clenbuterol in human saliva and urine. By optimizing the type and volume of extractant, pH of solution, volume of buffer, and type of aggregating agent, the final LOD of clenbuterol in saliva and urine was 25 ng/mL [73]. To further reduce the LOD, Peng’s team investigated the effect of metal nanoparticle type and particle size on the enhancement effect of clenbuterol, ractopamine, and salbutamol. The team configured aqueous solutions of clenbuterol, ractopamine, and salbutamol with different concentration gradients and explored the effects of different enhancement substrates, aggregating agents, and metal nanoparticle substrates with different particle sizes on the aqueous solutions of the three drugs. The results showed that the enhancement effect was the best for clenbuterol and ractopamine using gold nanoparticles as a substrate, and the enhancement effect was the best for salbutamol using silver nanoparticles as a substrate. As the particle size of the metal nanoparticles increased, the enhancement effect on the three drugs also increased. Under the optimal detection conditions, the limits of detection for the three drugs in an aqueous solution were 18 ng/mL, 24 ng/mL, and 0.2 mg/L, respectively [74,75]. On this basis, a quantitative prediction model was developed for clenbuterol levels in pork in the range of 1–10 µg/g with a correlation coefficient of 0.99 and a root mean square error of 0.263 µg/g [76]. It is well known that the SERS signal of clenbuterol does not increase indefinitely with the increasing particle size of gold nanoparticles. Therefore, the residues of clenbuterol in pork were detected using gold nanoparticles with larger particle sizes as a substrate. The results showed that the enhancement effect of clenbuterol was the best using gold nanoparticles as a substrate with 90 nm particle size. In addition, the team proposed that droplet size, shape, and laser-induced evaporation of the droplets affect the stability and reproducibility of the detection. Therefore, a sample collection assembly for the detection of clenbuterol was designed to solve the problem of poor reproducibility of SERS assay due to the difference in droplet shapes and sizes. The LOD of this method for clenbuterol was 42 ng/g [77]. Although the LOD was further reduced compared with the previous study, it was still at the same detection level.

The above methods require cumbersome extraction steps for detecting the residues of LMP in animal muscle tissues or urine, which cannot be performed directly. To address this problem, nanotips were formed on the surface by fabricating conical holes on an anodized aluminum template and through steps such as continuous etching and pore enlargement by Yan et al. Afterwards, silver nanoparticles were sputtered on the nanotips, and the bottom of the aluminum plate was removed to obtain a transparent SERS substrate consisting of ordered arrays of Ag NPs. The substrate can be directly applied to the surface of meat for the detection of ractopamine in pork with an LOD of 10^−8^ mol/L [78]. Although the preparation of the substrate is complicated, the method can be used without any pretreatment of pork for direct detection.

Most of the above papers used conventional spherical gold and silver nanoparticles as substrates for detecting the residues of LMP in animal tissues or urine. Although the LOD of LMP was reduced by optimizing the type, shape, size, and pH value of the gold and silver nanoparticles; the extraction method of target molecules, and the type of aggregating agent, the LOD always remained at the level of ng/mL or ng/g. There is still a certain distance from the national requirement of non-detectability and the LOD of traditional methods. In addition, the extraction of LMP in meat first, followed by detection, is destructive. The preparation of solid transparent substrates for directly detecting residues of LMP in meat has a greater development prospect.

#### 4.2.2. Metal Nanoparticles Bound to Adsorbent Materials

According to the EM mechanism, the enhancement effect of target molecules by metal nanoparticles mainly relies on the formation of “hot spots”. When more target molecules are located in the vicinity of the “hot spots”, the SERS signals will be stronger. Therefore, combining metal nanoparticles with adsorbent materials can enable the adsorption of target molecules, and target molecules located at “hot spots” can be aggregated, which can improve the SERS signals of the target molecules and reduce the LOD. Among the commonly used adsorbent materials are graphene oxide, aluminum trioxide, and *β*-cyclodextrin. Among them, graphene oxide shows remarkable adsorption capacity for metal ions and organic compounds through non-covalent interactions (including π–π interactions, van der Waals interactions, dispersive forces, hydrophobic effects, etc.) [104].

Gold nanoparticles were deposited on graphene oxide sheets to generate a high density of intrinsic “hot spots” among Au NPs by Sun et al., which were used as an enhancement substrate for the detection of aqueous clenbuterol solution. The enhancement of clenbuterol by this substrate was 4.8 times higher than that by ordinary gold nanoparticles due to the better adsorption of clenbuterol by graphene. The LOD of this method for clenbuterol was 3.34 × 10^−8^ mol/L [79]. Cheng et al. compared the enhancement effects of graphene oxide/gold nanoparticles (GO/AuNPs) and re-oxidized graphene/gold nanoparticles (roGO/AuNPs) used as enhancement substrates for the detection of salbutamol standard solutions, respectively. The results showed that salbutamol had the strongest bands at 808, 1145, 1264, 1437, and 1602 cm^−1^ when roGO/AuNPs were used as the enhancement substrate. Afterward, roGO/AuNPs were used as an enhancement substrate for the detection of spiked clenbuterol in urine. To exclude the effect of impurities in urine, carboxylic acid magnetic beads were combined with antibodies against salbutamol to prepare antibody-functionalized immunomagnetic beads (Ab-MBs), which were used to achieve the enrichment of salbutamol in the urine of animals through the specific binding of antibodies against salbutamol. The LOD of this method was 0.5 ng/mL with recoveries of 85.2–92.5% [80]. In addition, the team used the same method to detect clenbuterol in pig urine. The LOD for clenbuterol in pig urine was 0.5 ng/mL, the recoveries ranged from 82.8 to 92.4%, the coefficient of variation was <9.4%, and the detection time for each sample was less than 15 min [81]. As the enrichment of LMP using antibody-functionalized immunomagnetic beads (Ab–MBs) requires more complicated steps and expensive antibodies, the extraction method was improved. The mixture solvent di-(2-ethylhexyl) phosphate/methylene chloride (*v*/*v*, 1:800) was used as an extraction reagent, and the clenbuterol molecule was extracted from the urine into an aqueous solution by two liquid–liquid extractions and by adjusting the pH of the solution. Using melamine as the internal standard substance, the ratio of the characteristic bands related to clenbuterol and melamine was selected to establish a prediction model of clenbuterol content. The results showed that the method also achieved an LOD of 0.5 ng/mL for clenbuterol, with recoveries of 78.6–89.4% and coefficients of variation less than 9.8% [82]. Although the LOD was not reduced, the clenbuterol extraction step was simplified.

In summary, by combining metal nanoparticles with adsorbent materials, the LOD of LMP has been reduced by about 100 times compared to that of a single metal nanoparticle. This level has been much lower than the LOD set by the United States for ractopamine residues in meat, being at the same level as the traditional detection method.

#### 4.2.3. Metal Nanoparticles Bound to Chemical Substances

As meat contains more proteins, fats, and other complex interfering substances, although these substances can be removed by extraction, the residual impurities still have a great interference effect on the detection of LMP, which also restricts the sensitivity and stability of the SERS analysis. Therefore, combining the enhanced substrate with other chemicals, or indirectly through the method of chemical reaction, can greatly improve the precision and stability of the detection and reduce the LOD.

A highly ordered gold cavity array was prepared by electrodepositing gold nanoparticles onto the gaps of a polystyrene sphere template. Antibodies against salbutamol and trombuterol as well as DNA concatamers labeled with SERS reporter were immobilized on AuNPs to prepare immunoprobein, which was used to detect salbutamol and trombuterol in pork and pig liver with an LOD of 2.0 pg/mL and 1.0 pg/mL, respectively [83]. Compared to combining magnetic adsorbent materials, this method combined gold nanoparticles distributed spread on the solid substrate with antibodies, which reduces the LOD and improves the stability of the detection. An azo-coupling reaction with salbutamol was produced by adding sulfanilic acid and 2,20-benzidinedisulfonic acid to an alkaline solution to produce a color change, and the LOD for salbutamol by color was 5 µg/mL. Since the product of this azo reaction can increase the SERS cross-section (cross-section), Fe_3_O_4_@Ag nanoparticles were prepared to indirectly detect salbutamol with an LOD of 1.0 × 10^−10^ M [84]. The method avoids the use of expensive biological reagents and cumbersome and time-consuming sample pre-treatment processes and will enable the highly sensitive and selective detection of salbutamol using only a simple chemical reaction and color change. The whole process including pretreatment, coupling reaction, and SERS detection was completed within 7 min. Indirect detection of LMP by certain chemical reactions or by controlling the degree of reaction excludes the interference of impurities in the solution and may reduce the LOD. Nitrogen/silver-doped carbon dots (CDN/Ag) were prepared by Yao et al. that could promote the synthesis rate of gold nanoparticles, based on which the sensitive detection of clenbuterol was achieved by modifying antibody against clenbuterol on CDN/Ag for use as an immunoprobe. When synthesizing gold nanoparticles, a certain amount of Victoria blue B (VBB) was added as an internal standard substance. Competitive adsorption of clenbuterol to the antibody in solution can dissociate the nitrogen/silver-doped carbon dots (CDN/Ag), which promotes the synthesis of gold nanoparticles and thus enhances the SERS signal of VBB. The LOD of this method for clenbuterol was 0.68 pg/mL [85].

Extraction and enrichment of LMP from meat can effectively reduce the LOD. In addition, in all of the above reports, only one type of nanoparticle was used to detect the target molecules directly or indirectly, and the LOD level was kept at pg/mL and above. By simultaneous localized plasma resonance of two metal nanoparticles, the SERS signals of target molecules located at the common “hot spot” of the two particles can be greatly enhanced. In Duan’s report, the antibody against clenbuterol was modified on the surface of Fe_3_O_4_@Au@Ag NPs to prepare immunoprobes. Secondly, complementary DNA (cDNA) was modified on gold nanoparticles, carrying Raman reporter 4-mercaptobenzoic acid, to serve as a signaling probe. When the capture probe was combined with the signal probe, the Raman signal intensity could be significantly enhanced due to the dual coupling effect of Fe_3_O_4_@Au@Ag nanoparticles and Au nanoparticles. Therefore, when the concentration of clenbuterol was low, the signal probe was combined with the capture probe, and the SERS signal of the Raman reporter was enhanced. The LOD of this method for the residues of clenbuterol in pork was 3 pg/m [86]. Core–shell Fe_3_O_4_@Au nanoparticles were prepared by Wei et al. Antibodies against clenbuterol or salbutamol were modified on the surface of Fe_3_O_4_@Au@Ag NPs as magnetic probes. Utilizing the complementary function of antigen and antibody and the adsorption principle of magnetic field on Fe_3_O_4_, clenbuterol, or salbutamol was selectively separated. Subsequently, the matrix obtained after the reduction in the target concentration was analyzed using the SERS technique and it was determined that the separation method had better results [87]. The protocol was then optimized by the same group to include clenbuterol antibody-mercaptobenzoic acid-labeled Au as an immunoprobe and clenbuterol coating antigen-Fe_3_O_4_@Au nanoparticles as a clenbuterol-competitive immunoassay. After the entire reaction was completed, the precipitate was collected for SERS analysis using a magnetic field. In addition, due to the enhancement of Au and Fe_3_O_4_@Au dual nanoparticles, the SERS signal was greatly enhanced, and the LOD of this method for clenbuterol was 0.22 fg/mL [88]. The above literature combines magnetic nanomaterials with antibodies. Firstly, the target molecules are enriched by magnetic nanomaterials. After that, the enhancement by using bimetallic nanoparticles greatly increases the signal of SERS with an LOD level of fg/mL, which provides a new idea for the enhancement of target molecules using SERS substrates.

The LOD can also be reduced by utilizing the products of certain chemical reactions to affect the SERS signal. Immunoprobes were prepared using gold nanoparticles, catalase, and antibodies against ractopamine by Liang et al. Silver nanoparticles, ractopamine serum protein, and Raman reporter gene (4-mercaptobenzoic acid, 4-MBA) were used to prepare the capture probe. When hydrogen peroxide was added to the assay, the immunoprobe carrying catalase was progressively depleted as the concentration of ractopamine increased, so that the catabolism of hydrogen peroxide by catalase was reduced. The silver nanoparticles were reduced to silver by hydrogen peroxide and lost their enhancement of the Raman reporter gene (4-mercaptobenzoic acid, 4-MBA); thus, the Raman signal was reduced. The LOD of this method for ractopamine is 1 fg/mL [89]. Although this method has a high sensitivity for ractopamine, catalase is easily decomposed and unstable.

In summary, by combining nanoparticles with chemical substances, the LOD of LMP residues can be greatly reduced, and the detection level can reach pg/mL or even fg/mL. By detecting the internal standard substances or chemical reaction products, the interference of impurities in animal muscle tissues or urine can be reduced, and the reproducibility and stability of the test can be improved.

### 4.3. Application of SERS in Combination with Related Techniques for Detecting LMP in Meat

#### 4.3.1. SERS Combined with Immunochromatography

**Figure 4:**
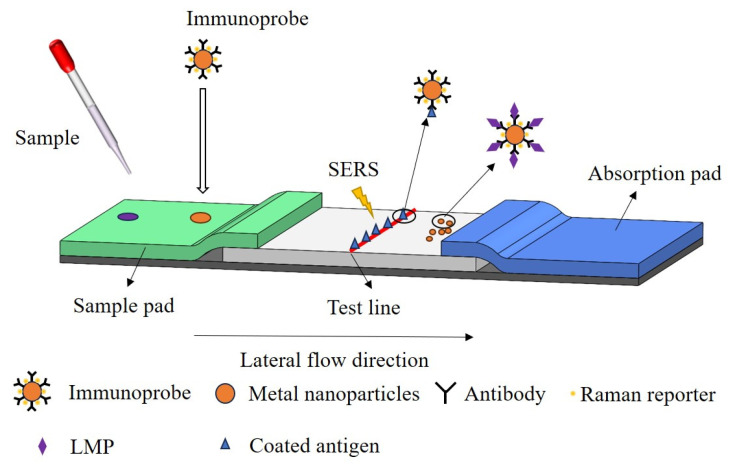
Schematic diagram of the process of detecting LMP by SERS-ICA.

Immuno–chromatography (ICA) is a membrane-based detection technique based on antigen–antibody interactions, which allows for the separation of reactive products from unreacted products without the need for additional precipitation or washing steps [105]. The specificity and sensitivity for detecting LMP in meat can be improved using the SERS in combination with the ICA. The schematic diagram of the process of detecting LMP by SERS–ICA is shown in Figure 4. First, the immunoprobes are prepared using metal nanoparticles, antibodies against LMP, and Raman reporters. Next, the sample to be measured and the immunoprobe are added dropwise to the sample pad of the immunoprobe paper, moving together to the right under the action of the absorption pad. LMP molecules in the samples will produce an immunoreaction with the immunoprobe. Probes not involved in the reaction are immobilized at the test line by being captured by the encapsulated antigen. The nanoprobes involved in the reaction continue to move to the right. Finally, the SERS spectra of the Raman reporter molecules on the test line are measured, resulting in an indirect prediction of LMP concentration. The concentration of LMP is inversely proportional to the intensity of the characteristic SERS peak.

In 2014, SERS combined with ICA was used to analyze phenylethanolamine A in porcine urine by Li et al. A Au^MBA^@Ag–Ab immunoprobe was prepared by immobilizing antibodies against phenylethanolamine A and a Raman reporter (4-mercaptobenzoic acid, MBA) on the surface of gold nanoparticles. A coated antigen (phenylethanolamine A bovine serum protein, PA-BSA) was applied to the test line of the test strip. During the detection, the sample to be tested was moved to the test line together with the immunoprobe. Since phenylethanolamine A bound to the immunoprobe first, as the concentration of phenylethanolamine A increased, the concentration of the immunoprobe captured by PA-BSA on the test line decreased, and the SERS signal of the Raman reporter, MBA, carried by the immunoprobe on the test line also decreased. The concentration of phenylethanolamine A was inversely proportional to the SERS intensity. Since MBA contains an independent characteristic band at 1074 cm^−1^ and has a high signal-to-noise ratio, this method reduces the influence of other impurities on the detection results. The LOD of this method for phenylethanolamine A was 0.32 pg/mL, and the whole process took about 15 min [90]. The LOD can be lowered by making changes to the reinforcement substrate. In 2015, an immunoprobe was prepared by Xie et al. using core–shell gold/silver nanoparticles instead of gold nanoparticles for the detection of clenbuterol in urine samples using the SERS–ICA technique. The LOD for clenbuterol by this method was 0.24 pg/mL [91]. The LOD was only slightly reduced compared to previous studies.

The probes are made by attaching antibodies or Raman reporter molecules to the surface of the gold nanoparticle shells, and the stability of the attachment affects the precision of the detection results. In 2017, flower-like core–shell gold and silver nanoparticles (AuNF@Ag) were prepared as enhancement substrates by Fu et al. The Raman reporter 4-mercaptobenzoic acid (MBA) was immobilized between the flower-like gold shells and the silver nuclei, and an antibody against brombuterol was immobilized on the surface of the gold shells as an immunoprobe for the detection of brombuterol in pork and urine with a detection line of 0.5 pg/mL. The method was selective for salbutamol, ractopamine, phenylethanolamine A, isoprenaline, and phenylephrine. However, the cross-reactivity with clenbuterol was 8.5%, which may be due to the high structural similarity. Although the LOD was not improved, the method has better stability due to the modification of MBA between the gold shell and silver core, which is not easy to dislodge. The recoveries of this method for the determination of brombuterol in pork and urine ranged from 95.8% to 108.0%, with relative standard deviations (RSDs) between 2.0 and 6.3% [92]. Su et al. chose 5,5′-dithiobis (2-nitrobenzoic acid) DTNB as a Raman reporter immobilized between the core (AuNP) and shell (Au nanostar). Antibodies against clenbuterol were physically adsorbed on the surface of gold nano-star shells to prepare immunoprobes. The LODs of clenbuterol standard solution by both eye viewing and SERS methods were 5 ng/mL and 0.05 ng/mL, respectively. The recoveries of this method for clenbuterol-spiked samples in pork were 86–110%. However, the method did not reduce the LOD, which may be due to the poor enhancement of the selected Raman reports by the star-shell nanostructures [93].

The SERS–ICA technique can also be used to detect two or more LMP drugs at the same time. An immunoprobe was reported by Yu et al., which consisted of 4,40-dipyridyl (DP), the antibody against clenbuterol, 2,20-dipyridyl (BP), the antibody against ractopamine, and gold nanoparticles, based on the SERS and multiple competitive immunoassay methodology to achieve the simultaneous detection of two drugs, clenbuterol and ractopamine. Clenbuterol bovine serum protein and ractopamine bovine serum protein were simultaneously modified and immobilized on concave glass substrates. Therefore, when the clenbuterol and ractopamine standard solution and the prepared immunoprobes are added to the substrate dropwise at the same time, the clenbuterol and ractopamine in the solution will compete with the serum proteins on the substrate to undergo an immunosorbent reaction with the antibodies on the immunoprobes. Finally, the simultaneous detection of clenbuterol and ractopamine is realized by measuring the SERS spectra of the residues on the substrate. In the detection, clenbuterol bovine serum protein and ractopamine bovine serum protein were immobilized at different positions on the substrate to avoid cross-reactivity. Therefore, the two characteristic bands can be used to identify and quantitatively predict the types of added samples at the same time. The method can realize the simultaneous detection of clenbuterol and ractopamine in a wide concentration range of 1–1000 pg/mL with an LOD of 1 pg/mL [94]. This method employed two Raman reporters for the immobilization of two drug antibodies respectively, which are more complicated to prepare. Ractopamine, clenbuterol antibodies, and MBA were immobilized in core–shell Fe_3_O_4_@Au nanoparticles to prepare immunoprobes by Wu et al., which made the preparation of nanoprobes much simpler. Simultaneous detection of ractopamine and clenbuterol in pork, beef, and lamb was realized based on the SERS–ICA method. The LODs were 1 and 0.33 ng/mL based on the colorimetric method and 7.8 and 3.5 pg/mL based on SERS, respectively [95].

The quantitative analysis of SERS is always affected by the colloid size and the degree of aggregation of colloidal particles. To solve this problem, a multilayer magnetic core double-shell nanoparticle (MDAu@Ag) was prepared with Fe_3_O_4_ as the core and gold and silver as the bilayer shell by Tu et al. The advantage of this nanoparticle was that the gap between the gold and silver double shells was consistent, and by immobilizing the two Raman reporter molecules between the gold and silver double shells, the stability of the SERS signal was greatly improved. This method allowed simultaneous and direct detection of four drugs, clenbuterol, ractopamine, chloramphenicol, and kanamycin, in ambient lake water, as well as in simple extracts of pork and feed. The LODs of this method were 6.2 and 2.5 pg/mL for clenbuterol and ractopamine, respectively, with recoveries ranging from 91.63 to 112.74% and 87.91 to 114.72%, respectively [96].

All of the above reports detected residues of LMP in animal muscle tissues or urine, etc. However, muscle tissue and urine are not easily acquired and preserved. This problem can be well solved by detecting the residues of LMP in animal hair, which are evenly distributed in the hair and can be traced over time. SERS–ICA was used to detect clenbuterol in pig hair extracts by Zheng et al., who fabricated a novel 3D paper microfluidic device that can be used to control the interactions between different microfluidic fluids by manually pulling the slides, which makes the SERS/immunoassay operation simpler and management of the samples easier. The LOD of this method for clenbuterol in pig hair extract was 0.1 pg/mL [97]. A 3D microfluidic paper coupled with the SERS method to measure clenbuterol, ractopamine, and salbutamol in pig hair extracts was reported by Dou et al. This 3D microfluidic paper contained two layers of filtration area and detection area. For the detection, the microphase paper was folded to bring the filtration and assay areas into contact. Afterwards, the hog hair extract was dropped into the filtration region. After the liquid in the assay region was dried by hot airflow within a few seconds, the up-sampling process was repeated 20 times to allow the LMP to accumulate in the assay region. Finally, a gold nanoparticle (AuNP) solution was dropped into the detection region to collect SERS spectra. During the detection process, macromolecular impurities such as pig hairs were filtered in the re-filtration zone and the target molecules entered the detection zone. Target molecules were enriched in the detection zone by repeated sampling and drying. Finally, the target molecules were detected by the SERS technique, which had an LOD of ng/mL [98]. This method can filter the sample and reduce the sample pretreatment process, which makes the operation much simpler.

The LOD of LMP residues in meat was at the level of pg/mL using SERS-ICA, which can detect LMP in meat by both color qualitative and SERS quantitative routes. Furthermore, without the need for a precise extraction process, the detection process is relatively simple. However, the preparation of nanoprobes is complicated, and the use of different metal nanoparticles to make nanoprobes has a certain effect on the LOD. In addition, the connection between the Raman report and the metal nanoparticles is easily detached, which affects the stability of the detection results and the detection accuracy. It is crucial to make the nanoprobes more stable, and to select Raman reporter molecules that respond better to SERS signals.

#### 4.3.2. SERS in Combination with Other Techniques

SERS with an immunoassay (IA) was combined to achieve the detection of clenbuterol in porcine urine by Zhu et al. In this report, nanoparticle immunoprobes of Au^DP^–Ab were prepared, and clenbuterol bovine serum proteins (antigens) were immobilized on gravure plates. After immune competition of clenbuterol with antigen against antibody, the reactants were washed away and the residues were detected. The LOD of this method for clenbuterol was 0.1 pg/mL [99]. Compared with the reports of SERS–ICA in the detection of LMP, the LOD was not greatly improved, but there was an additional step of washing the substrate, which complicates the detection process. Molecularly imprinted solid-phase extraction with the SERS technique was used to detect ractopamine in pork tissues by Xiao et al. Ritodrine was used as a pseudo-template molecule to synthesize molecularly imprinted polymers (MIPs), which showed better selectivity for ractopamine than ritodrine. Based on this principle, MIPs were used as adsorbents for the selective enrichment of ractopamine for solid-phase extraction, and then gold nanoparticles were used as the reinforcing substrate for the detection of ractopamine, with an LOD of 3.1 µg/L and recoveries of 72.4–79.7% [100]. Although the LOD of this method was high, it used molecularly imprinted polymers instead of organic solvents, which reduced the use of organic solvents and is more friendly to the environment. A SERS/electrochemical dual-signal readout immunosensor was established by Gu et al. The sensor prepared a highly ordered gold/silver bimetallic cavity array (BMCA) by electrodepositing Au/Ag nanoparticles into the interstices of a highly ordered close-packed polystyrene template. Based on this sensor, salbutamol, ractopamine, and phenylethanolamine A can be detected by both SERS and electrochemical methods, with the lowest LOD for the quantitative analysis of the three drugs using the SERS method. The limits of detection were 0.8, 0.4, and 1.3 pg/mL with recoveries ranging from 95.0% to 108.5%, respectively [101]. This report prepared nano-sensors that can simultaneously employ SERS and electrochemical techniques for the detection of LMP molecules, but it did not fuse the information from both for data analysis to reduce the LOD.

## 5. Conclusions and Future Trends

In summary, the LOD of LMP residues in meat using the SERS technique is in the range of fg/mL, which indicates that the SERS technique has great potential for the detection of LMP residues in meat. Due to the limitation of the sensitivity of the current detection equipment, the LOD with the enhancement of aqueous solution of LMP by a single gold nanoparticle or silver nanoparticle is high, and it cannot yet be used for practical detection. However, by combining the metal nanoparticles with adsorbent materials or chemicals such as immunizations, the LOD can be as low as ng/mL. In addition, the SERS signals of LMP molecules were greatly enhanced and the LOD was as low as fg/mL when the target molecules were detected by coupling two metal nanoparticles. However, the use of simple SERS labeling including colloidal nanoparticles or Au- or Ag-coated core–shell structured nanoparticles for detecting residues of LMP in meat still suffers from the problems of poor stability of complex samples, insufficient SERS activity, and uncontrolled “hot spots”, which make it difficult to commercialize and standardize this method. When SERS technology is combined with other technologies to detect LMP, although the detection results are more stable and the LOD reaches ng/mL, there are still problems such as the preparation of nanoprobes being more complicated, the antibody being more expensive, and the detection only being able to be carried out on a single or a specific species of drugs. In the future, the SERS technique instead of the traditional technique for detecting the residues of LMP in meat should be explored in the following aspects for lower LOD and accurate and stable measurement results.

(1) Development of novel enhancement substrates with larger SERS enhancement factors and stabilized SERS signals will greatly enhance the SERS signal.
(2) Deposition of metal nanoparticles on a densely ordered solid substrate results in a more uniform “hot spot” produced by the metal nanoparticles.
(3) Combining metal nanoparticles with physically or chemically adsorbent materials allows for better adsorption of target molecules on the surface of the nanoparticles, resulting in higher SERS intensities and stable signals.
(4) Development of solid transparent substrates to reduce the extraction process of LMP residues in meat and to detect them directly on the meat, thus realizing non-destructive and rapid detection.
(5) The combination of two or more nanoparticles for the simultaneous detection of target molecules can greatly enhance the SERS signal of target molecules.
(6) More information on SERS spectra should be mined. Currently, it has been reported that the characteristic bands of SERS signals are mainly selected to establish a ULR, MLR, or PLSR model with the concentration of substances, and the modeling process is relatively simple. The information on the characteristic bands is underutilized, such as the shape of the characteristic bands, the area, etc. The prediction of residual concentrations of target molecules using neural network modeling will be the future direction.

## Figures and Tables

**Table 1:** Applications of SERS spectroscopy for the detection of LMP in meat.

Method	Enhanced Substrate	Material	Target Molecule	Quantitative Range	LOD	References
SERS	-	Rat meat	Clenbuterol	5–10,000 ppb	–	[67]
SERS	MAgNPs	Water	Salbutamol	0–1000 ng/µL	1.25 ng/μL	[68]
SERS	Au NPs	Water	Clenbuterol, salbutamol, terbutaline	0–10^−4^ mol/L	35, 765, 55 ng/mL	[69]
SERS	Au NPs	Water	Clenbuterol, ractopamine	0.1–5 mg/L	0.1 mg/L	[70]
SERS	Au NPs	Standard solution	Clenbuterol, ractopamine, salbutamol,	1–20 mg/L	0.002, 0.1, 0.002 mg/L	[71]
SERS	Gold-plated Klarite	Swine urine	Ractopamine,	0.4–8 µg/mL	0.4 μg/mL	[72]
SERS	Ag NPs	Saliva, urine	Clenbuterol	2.5–1000 ng/mL	25 ng/mL	[73]
SERS	Ag NPs	Standard solution	Salbutamol	0.2−1 mg/L	0.2 mg/L	[74]
SERS	Au NPs	Standard solution	Clenbuterol, ractopamine	0.1–1 µg/mL	18 ng/mL, 24 ng/mL	[75]
SERS	Au NPs	Pork	Clenbuterol	1–10 µg/g	1 µ g/g	[76]
SERS	Au NPs	Pork	Clenbuterol	0.5–15 µg/mL	42 ng/g	[77]
SERS	Ag NPs	Pork	Ractopamine	10^−8^−10^−4^ M	1.0 × 10^−8^ M	[78]
SERS	GO/AuNP	Standard solution	Clenbuterol	5 × 10^−8^–10^−6^ mol/L	3.34 × 10^−8^ mol/L	[79]
SERS	RoGO/AuNPs	Animal urine	Salbutamol	1–20 ng/mL	0.5 ng/mL	[80]
SERS	Au NPs	Swine urine	Clenbuterol	0.5–20 ng/mL	0.5 ng/mL	[81]
SERS	roGO/AuNPs	Animal urine	Clenbuterol	1–20 ng/mL	0.5 ng/mL	[82]
SERS	GCA-Ab	Pork, pig liver, urine	Salbutamol, trombuterol	0.005–100, 0.003–200 ng/mL	1.0, 2.0 pg/mL	[83]
SERS	Fe_3_O_4_@Ag	Pork	Salbutamol	10^−11^–10^−6^ M	1.0 × 10^−10^ M	[84]
SERS	Au NPs	Standard solution	Clenbuterol	0.0033–0.067 ng/mL	0.68 pg mL	[85]
SERS-IA	Fe_3_O_4_@Au@Ag	Pork	Clenbuterol	0.01–10 ng/mL	3 pg/mL	[86]
SERS-IA	Fe_3_O_4_@Au-Ab	Standard solution	Clenbuterol, salbutamol	-	17 fg/mL	[87]
SERS-IA	Fe_3_O_4_@Au-Ab	Standard solution	Clenbuterol	1–100 ng/mL	0.22 fg/mL	[88]
SERS-Elisa	Ag NPs	Swine urine	Ractopamine	-	10^−6^ ng/mL	[89]
SERS-IAC	Au^MBA^ @Ag-Ab	Swine urine	Phenylethanolamine A	0–100 ng/mL	0.32 pg/mL	[90]
SERS-ICA	Au^MBA^ @Ag–Ab	Swine urine	Clenbuterol	0–10 ng/mL	0.24 pg/mL	[91]
SERS-LFIA	AuNFs^MBA^@Ag-Ab	Pork, swine urine	Brombuterol	0–100 ng/mL	0.5 pg/mL	[92]
SERS-LFIA	Au@Au nanostar	Standard solution	Clenbuterol	0.05–1 ng/mL	0.05 ng/mL	[93]
SERS-	Ab–^BP^Au^DP^–Ab	Standard solution	Clenbuterol, ractopamine	1–1000 pg/mL	1 pg/mL	[94]
SERS-IAC	Fe_3_O_4_@Au-Ab	Pork, beef, and lamb	Clenbuterol, ractopamine	0–3 ng/mL	7.8 pg/mL 3.5 pg/mL	[95]
SERS-LFA	Fe_3_O_4_@Au@Ag	Pork, lake water	Ractopamine, clenbuterol	-	2.5, 6.2 pg/mL	[96]
SERS	Au^MBA^-Ab	Pig hair	Clenbuterol	0.1–100 pg/mL	0.1 pg/mL	[97]
SERS	Ag NPs	Pig hair	clenbuterol, ractopamine, salbutamol	-	20, 20, 30 ng/mL	[98]
SERS-IA	Au NPs-Ab	Swine urine	Clenbuterol	0.1–100 pg/mL	0.1 pg/mL	[99]
SERS	Au NPs	Pork, pig liver	Ractopamine	20.0–200.0 μg/L	3.1 μg/L	[100]
SERS	Au/Ag NPs	Pig liver	Salbutamol, ractopamine, phenylethanolamine A	0.002–200 0.001–200 0.005–100 ng/mL	0.8, 0.4, 1.3 pg/mL	[101]

## Data Availability

Not applicable.
